# Pharmacokinetics and bioequivalence of Ezetimibe tablet versus Ezetrol®:an open-label, randomized, two-sequence crossover study in healthy Chinese subjects

**DOI:** 10.1186/s40360-023-00649-y

**Published:** 2023-02-03

**Authors:** Feifei Sun, Yanping Liu, Ting Li, Pingping Lin, Xin Jiang, Xin Li, Chenjing Wang, Xiaomeng Gao, Yaping Ma, Yao Fu, Yu Cao

**Affiliations:** Phase I Clinical Research Center, The Affiliated Hospital of Qingdao University, Qingdao, 266003 Shandong China

**Keywords:** Ezetimibe tablet, Ezetimibe glucuronide, Bioequivalence, Pharmacokinetics, Healthy volunteers

## Abstract

**Background:**

Ezetimibe is a new class of antihyperlipidemic agent indicated for the prevention of atherosclerosis disease and for the treatment of hypercholesterolemia. Information on the pharmacokinetic profiles of ezetimibe tablet in healthy Chinese volunteers are lacking, and regulatory requirements necessitate a bioequivalence study of ezetimibe tablet versus Ezetrol® in China.

**Methods:**

A single-dose randomized, open-label, two-group, two-period crossover study was conducted in 59 healthy Chinese volunteers under fasting or fed conditions to assess the bioequivalence between two preparations. Eligible participants were randomly divided into fasted and fed groups. Blood samples were collected at specified time intervals, and the plasma concentrations of ezetimibe and ezetimibe glucuronide were determined by a validated liquid chromatography-tandem mass spectrometry (LC–MS/MS) method. PK and bioavailability parameters were estimated via non-compartmental methods. Adverse events were also recorded.

**Results:**

Fifty-nine healthy volunteers were enrolled in the study. The main pharmacokinetic parameters of total ezetimibe in the plasma of the ezetimibe tablet (10 mg) and the Ezetrol® (10 mg) after a single fasting administration: C_max_ were (65.73 ± 47.14), (71.32 ± 51.98) ng·mL^− 1^; T_max_ were 1.75, 1.25 h; T½ were (17.09 ± 13.22), (17.35 ± 12.14) h; AUC_0-t_ were (643.34 ± 400.77), (668.49 ± 439.57) h·ng·mL^− 1^; AUC_0-∞_ were (706.36 ± 410.92), (734.23 ± 468.26) h·ng·mL^− 1^. The main pharmacokinetic parameters of total ezetimibe in plasma of ezetimibe tablet (10 mg) and Ezetrol® (10 mg) after a fed administration: C_max_ were (83.38 ± 38.95), (84.74 ± 34.62) ng·mL^− 1^; T_max_ were 2.50, 2.50 h; T½ were (22.56 ± 12.68), (19.80 ± 15.59) h; AUC_0-t_ were (494.21 ± 208.65), (536.69 ± 209.11) h·ng·mL^− 1^; AUC_0-∞_ were (573.74 ± 252.74), (604.75 ± 247.13) h·ng·mL^− 1^. The main pharmacokinetic parameters C_max_, AUC_0-t_, and AUC_0-∞_ of the two drugs were analyzed by variance analysis after logarithmic transformation. The total ezetimibe under fasting state with 90% confidence intervals (CIs) were 85.29 ~ 97.19, 90.41% ~ 104.38%, and 90.81 ~ 106.05%; total ezetimibe in fed state were 86.36% ~ 109.17, 84.96% ~ 96.40, and 85.32% ~ 101.0%. The 90% CIs of the ratio of geometric means (GMRs) of C_max_, AUC_0-t_, and AUC_0-∞_ of Ezetrol® and ezetimibe tablet both fasting and fed conditions fell within the conventional bioequivalence criteria of 0.80–1.25. Both C_max_ and AUC met the predetermined criteria for assuming bioequivalence. No severe adverse events were observed.

**Conclusions:**

The test ezetimibe tablet and Ezetrol® were determined to be bioequivalent under both fasting and fed conditions in Chinese people.

**Trial registration:**

Clinicaltrials, NCT05681247 (retrospectively registered in 11/01/ 2023).

## Background

Ezetimibe (Ezetrol), as a non-statin lipid-lowering approach, was approved by US Food and Drug Administration (FDA) in patients with hypercholesterolemia (https://en.wikipedia.org/wiki/Ezetimibe, (https://pubchem.ncbi.nlm.nih.gov/compound/Ezetimibe#section=Top, https://www.drugbank.ca/drugs/DB00973, [1, 2]) Ezetimibe, administered in combination with a 3-hydroxy-3-methylglutaryl-coenzyme A (HMG-CoA) reductase inhibitor (statin), is indicated as adjunctive therapy to diet for the reduction of elevated total-C, LDL-C, Apo B, and non-HDL-C in patients with primary (heterozygous familial and non-familial) hyperlipidemia ([3–5], https://dailymed.nlm.nih.gov/dailymed/drugInfo.cfm?setid=A773B0B2-D31C-4FF4-B9E8-1EB2D3A4D62A) Ezetimibe reduces blood cholesterol by inhibiting the absorption of cholesterol by the small intestine. It takes effect through acting at the brush border of the small intestine and inhibiting the absorption of cholesterol, which leading to a decrease of hepatic cholesterol stores and an increase in clearance of blood cholesterol. After oral administration, ezetimibe is rapidly and extensively metabolized in the intestinal wall and the liver to a corresponding phenol glucuronide. This glucuronide is reexcreted in the bile back to its active site [6].

In this study, it is meaningful to investigate the pharmacokinetic characteristics and bioequivalence of single-dose ezetimibe tablets and Ezetrol® in Chinese healthy subjects under fasting and fed conditions.

## Methods

### Ethics approval

The trial was performed abiding by the Declaration of Helsinki [7], Good clinical practice (GCP) [8] and the guidelines of China National Medical Products Administration (NMPA). Relevant documents were all approved independently by the Medical Ethics Committee of the Affiliated Hospital of Qingdao University (Ethics approval No. QYFYEC 2017–026-01). Written informed consent was obtained from all subjects before their participation in the study. All protocol violations have been reported to the Medical Ethics Committee.

### Subjects

Chinese males and females aged 18 and above with a body mass index in the range of 18.0–26.0 kg/m^2^ (including the boundary value) were eligible for inclusion. The body weight of males and females was at least 50 kg and 45 kg, respectively. Serum total cholesterol was between 2.9 and 5.0 mmol/L (not including the boundary value). All volunteers were healthy, as confirmed by medical history, physical examination, vital sign measurements, 12-lead ECG and laboratory safety tests including hematology and urinalysis. The exclusion criteria included as follows: patients with medical history of cardiovascular, digestive, respiratory, nervous or hematological diseases, abnormal vital signs, allergic to ezetimibe or its excipients, lactating or pregnant women,drug or alcohol abuse, smoking≥5 cigarettes per day, donation (≥300 ml) or enrollment in other clinical trials within 3 months, any use of prescription drugs or vitamins or caffeine/xanthine-rich beverages 48 h prior to taking medication.

### Study design

This was a randomized, single center, open-label, single-dose, two-period crossover phase I clinical trial designed to enroll healthy volunteers with the aim of comparing PK parameters of Ezetrol® (10 mg, batch no.5 EZPA42002, from MSD Pharma (Singapore) Pte. Ltd.) to those of ezetimibe tablet (10 mg, batch no. 170301, from Chongqing shenghuaxi Pharmaceutical Co., Ltd.). All study drugs were provided by the sponsor at no cost.

Randomization was planned to generate two subject groups (fasting conditions and fed conditions) with administration of the products in two stages, separated by a 14-day washout period. Each eligible subject will be given a randomization number from small to large according to the screening number.

The qualified volunteers were hospitalized in the phase I clinical research center on the day before dosing and placed on a uniform diet during hospitalization. The fasting group were fasted for overnight (10 h) before administration. The fed group was requested to take a high-fat breakfast (total energy 1000 cal, 65% fat, 15% protein, 20% carbohydrate) within 0.5 h before administration. The management provided standard lunch and dinner for participants at 4 h and 10 h after dosing, separately. They then returned to the facility for regular blood sampling up to a total duration of 14 days. Blood samples (4 ml) were collected in precooled vacuum tubes containing sodium citrate anticoagulant in each stage (pre-administration and at 0.16, 0.3, 0.5, 1, 1.5, 2, 2.5, 3, 4, 5, 6, 8, 10, 12, 24, 36, 48, 72 h after administration).

The plasma samples were centrifuged at 4000 g for 10 min at 4 °C and future stored at − 80 °C. Bio-analysis experiment was analyzed by LC–MS/MS methods at Chongqing shengshifuma Technology Co., Ltd, [9]. Accuracy expressed as bias ranged from − 0.7 to 1.8% and 0.7 to 3.5% for ezetimibe and ezetimibe glucuronide. The lower limit of ezetimibe and ezetimibe glucuronide were 0.15 ng/mL and 1.5 ng/mL respectively.

### Safety and tolerability evaluations

The tolerability and safety of ezetimibe tablet were evaluated based on adverse-event reports, physical examinations, vital signs (body temperature, blood pressure and heart rate), 12-lead ECG and clinical laboratory tests (serum chemistry, hematology and urinalysis). Vital signs, were measured in each stage (screening, pre-administration and at 1, 4, 12, 24, 48, 72 h after administration). Routine laboratory tests and 12-lead ECG were conducted at screening and before removal from the study (18 days). Adverse events (AEs) were monitored throughout the study and were graded in accordance with the Common Terminology Criteria for Adverse Events (CTCAE) version 4.03.

### Pharmacokinetic analysis

Pharmacokinetic parameters of ezetimibe and ezetimibe glucuronide were calculated with DAS 3.3.1 software by noncompartmental analysis method. The primary PK parameters were the maximum plasma concentration (C_max_), the area under the plasma concentration-time curve from 0 to the last measured time point (AUC_0-t_), and the area under the plasma concentration-time curve from 0 to infinity (AUC_0-∞_). The secondary PK parameters were the observed time to C_max_ (T_max_) and the apparent terminal half-life (T½). C_max_ and T_max_ were obtained from the date and AUC_0-t_ was calculated using the linear and logarithmic trapezoidal rule. AUC_0-∞_ was calculated as the sum of AUC_0-t_ and C_last_/λ_z_ (C_last_ is the last measurable concentration and λ_z_ is the slope of linear regression after logarithmic conversion at the end of the concentration–time curve). T½ was calculated to be ln2/λ [10].

### Statistical analysis

Statistical analysis was performed by SAS 9.4 (SAS Institute Inc. Cary, NC, USA). Demographic characteristics, safety parameters and PK data were summarized using descriptive statistics,.Statistical data were presented as mean ± standard deviation (SD) The differences between groups were determined by two one-sided tests [10]. The probability value less than 0.05 was considered statistically significant.

Analysis of variance (ANOVA) was performed on the logarithmically transformed C_max,_ AUC_0-t_ and AUC_0-∞_. A mixed-effect model that included treatment group, period and formulation as fixed effects and subject within sequence as random effect was used for all comparisons. The geometric mean ratios (GMRs) ofthe primary PK parameters and their 90% confidence intervals (CIs) were calculated. The two preparations were considered bioequivalent if the 90% CIs of GMRs of the primary PK parameters were within the predefined acceptance range of 80–125%.

## Results

### Subject characteristics

In fasting group, 30 randomized participants (28 males and 2 females) enrolled and completed the study. The demographic details, mean ± SD (range): age, 27.83 ± 6.25 years (18.0–38.0 years); height, 170.25 ± 5.79 cm (159–179.5 cm); weight, 62.87 ± 7.88 kg (46–77 kg); body mass index (BMI), 21.67 ± 2.3 kg/m^2^ (18.1–26 kg/m^2^). In fed group, 29 randomized participants (21 males and 8 females) enrolled and completed the study. One subject voluntarily withdrew before high-fat meal, and the other subject withdrew due to intolerance to high-fat meal. The demographic details, mean ± SD (range): age, 25.72 ± 6.52 years (18–44 years); height, 167.9 ± 7.81 cm (153–182 cm); weight, 61.66 ± 7.82 kg (48–77 kg); BMI, 21.87 ± 2.31 kg/m^2^ (8.6–26 kg/m^2^). Thus, all subjects who received study drugs were included in the safety analysis set and PK analysis set.

### Pharmacokinetics

**Fig. 1:**
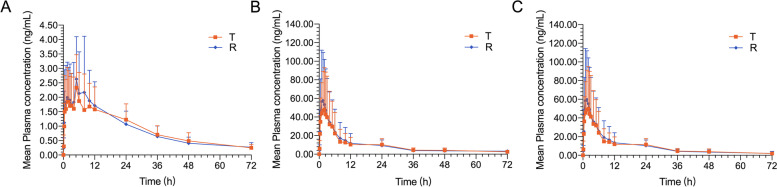
Mean plasma concentration versus time profiles of ezetimibe (**A**), ezetimibe glucuronide (**B**), and total ezetimibe (**C**) under fasting conditions, following a single dose of the test (T, 10 mg ezetimibe tablets) and reference (R, 10 mg Ezetrol®) in Chinese subjects. Error bars represent standard deviations, *n* = 30

**Fig. 2:**
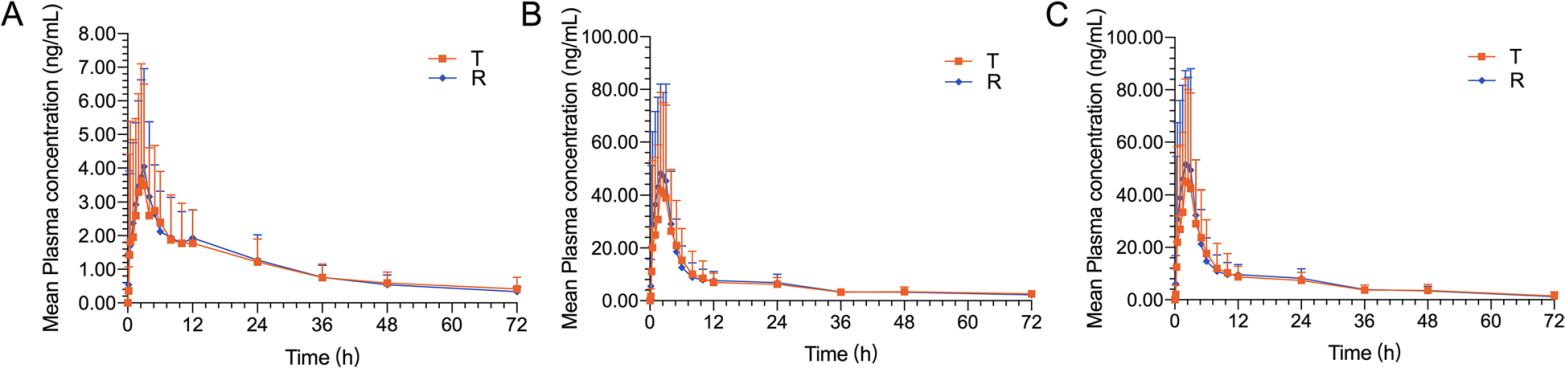
Mean plasma concentration versus time profiles of ezetimibe (**A**), ezetimibe glucuronide (**B**), and total ezetimibe (**C**) under fed conditions, following a single dose of the test (T, 10 mg ezetimibe tablets) and reference (R, 10 mg Ezetrol®) in Chinese subjects. Error bars represent standard deviations, *n* = 29. a Mean plasma concentration-time curves of ezetimibe. b Mean plasma concentration-time curves of ezetimibe glucuronide .c Mean plasma concentration-time curves of total ezetimibe

**Fig. 3:**
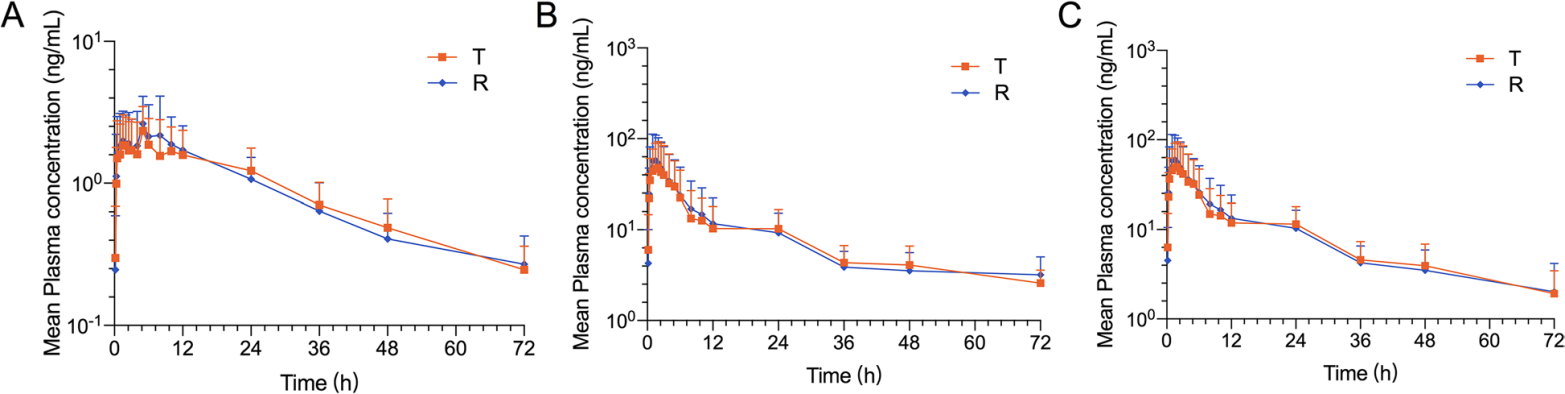
Semi-logarithmic curves of mean plasma concentration versus time profiles of ezetimibe (**A**), ezetimibe glucuronide (**B**), and total ezetimibe (**C**) under fasting conditions, following a single dose of the test (T, 10 mg ezetimibe tablets) and reference (R, 10 mg ezetrol®) in Chinese subjects. *n* = 30

**Fig. 4:**
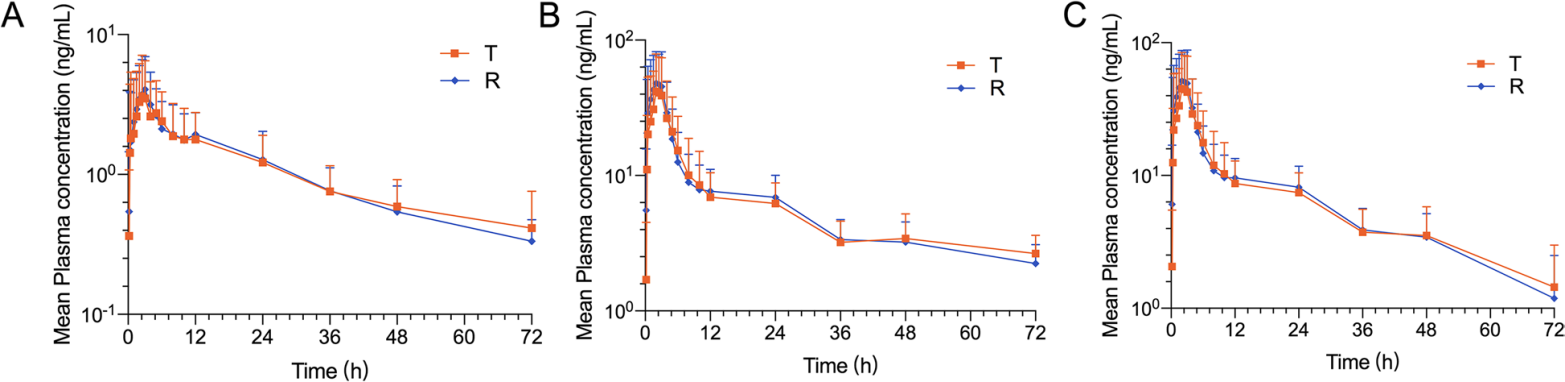
Semi-logarithmic curves of mean plasma concentration versus time profiles of ezetimibe (**A**), ezetimibe glucuronide (**B**), and total ezetimibe (**C**) under fed conditions, following a single dose of the test (T, 10 mg ezetimibe tablets) and reference (R, 10 mg ezetrol®) in Chinese subjects. *n* = 29

All subjects completed the study and the data were included in the pharmacokinetic analysis. The mean plasma concentration versus time profiles of ezetimibe and ezetimibe glucuronide under fasting and fed conditions are illustrated in Figs. 1,2,3 and 4. The pharmacokinetic parameters were summarized in Table 1. The 90% CIs for the geometric mean ratios (GMRs) of C_max_, AUC_0–t_, and AUC_0-∞_, and the power under fasting and fed conditions were presented in Table 2.

**Table 1:** The PK parameters of ezetimibe, ezetimibe glucuronide, and total ezetimibe under fasting and fed conditions

	Fasting group		Fed group	
	T	R	T	R
**Ezetimibe**				
C_max_ (ng/mL)	3.23 ± 1.41	3.48 ± 1.82	5.94 ± 4.12	5.81 ± 3.06
AUC_0–t_ (ng·h/mL)	58.49 ± 25.76	59.52 ± 27.21	72.67 ± 36.74	76.0 ± 35.48
AUC_0–∞_ (ng·h/mL)	68.05 ± 28.60	68.62 ± 30.60	88.50 ± 43.95	88.98 ± 38.33
T_max_ (h)	5.0 (0.50–24.0)	5.0 (0.33–12.0)	2.50 (0.50–6.0)	2.50 (0.33–5.0)
T_1/2_ (h)	18.03 ± 6.52	19.38 ± 11.41	25.89 ± 12.01	26.62 ± 15.12
λ_z_ (h^− 1^)	0.043 ± 0.015	0.048 ± 0.024	0.033 ± 0.018	0.033 ± 0.015
**Ezetimibe glucuronic acid**				
C_max_ (ng/mL)	63.21 ± 47.14	68.55 ± 51.82	77.68 ± 37.25	79.22 ± 33.05
AUC_0–t_ (ng·h/mL)	575.78 ± 408.26	598.91 ± 441.88	409.29 ± 191.89	445.64 ± 196.37
AUC_0–∞_ (ng·h/mL)	652.95 ± 407.06	690.65 ± 456.82	532.09 ± 214.84	550.38 ± 214.12
T_max_ (h)	1.75 (0.33–5.0)	1.50 (0.33 ~ 5.0)	2.50 (0.50–6.00)	2.50 (0.50–5.0)
T_1/2_ (h)	20.36 ± 11.62	22.57 ± 21.67	31.05 ± 17.04	27.78 ± 13.71
λ_z_ (h^− 1^)	0.049 ± 0.041	0.051 ± 0.036	0.028 ± 0.015	0.030 ± 0.012
**Total ezetimibe**				
C_max_ (ng/mL)	65.73 ± 47.14	71.32 ± 51.98	83.38 ± 38.95	84.74 ± 34.62
AUC_0–t_ (ng·h/mL)	643.34 ± 400.77	668.49 ± 439.57	494.21 ± 208.65	536.69 ± 209.11
AUC_0–∞_ (ng·h/mL)	706.36 ± 410.92	734.23 ± 468.26	573.74 ± 252.74	604.75 ± 247.13
T_max_ (h)	1.75 (0.33–5.0)	1.25 (0.33–5.0)	2.50 (0.50–5.0)	2.50 (0.50–6.0)
T_1/2_ (h)	17.09 ± 13.22	17.35 ± 12.14	22.56 ± 12.68	19.80 ± 15.59
λ_z_ (h^− 1^)	0.062 ± 0.042	0.061 ± 0.033	0.041 ± 0.022	0.052 ± 0.027

*Abbreviation*: *C*_*max*_ Maximum concentration, *AUC*_*0-t*_ The area under the plasma concentration-time curve from 0 h to the time of last measurable concentration, *AUC*_*0-∞*_ The area under the plasma concentration-time curve from 0 h to infinity, *T*_*max*_ Time to reach maximum concentration (Median), *T½* half life, *λ*_*z*_ terminal elimination rate

**Table 2:** The geometric mean ratios of primary pharmacokinetic parameters for ezetimibe, ezetimibe glucuronide, and total ezetimibe and their 90%CIs under fasting and fed conditions

	Geometric mean		T/R(%)	Intra-CV(%)	90%CIs
	T	R			
**Ezetimibe, fasting**					
C_max_ (ng/mL)	2.96	3.12	94.89	21.71	86.26 ~ 104.38
AUC_0-t_ (ng·h/mL)	53.59	54.93	97.56	20.81	89.04 ~ 106.9
AUC_0-∞_(ng·h/mL)	62.48	63.69	98.09	20.19	89.77 ~ 107.19
**Ezetimibe glucuronide, fasting**					
C_max_ (ng/mL)	52.02	56.98	91.30	15.28	85.37 ~ 97.63
AUC_0-t_ (ng·h/mL)	469.63	486.21	96.59	18.22	89.16 ~ 104.64
AUC0-∞(ng·h/mL)	551.63	575.92	95.78	15.84	89.35 ~ 102.68
**Total ezetimibe, fasting**					
C_max_ (ng/mL)	54.69	60.07	91.05	14.87	85.29 ~ 97.19
AUC_0-t_ (ng·h/mL)	550.93	567.13	97.14	16.35	90.41 ~ 104.38
AUC_0-∞_(ng·h/mL)	610.67	622.30	98.13	17.66	90.81 ~ 106.05
Ezetimibe, fed					
C_max_ (ng/mL)	4.92	5.11	96.28	24.53	86.27 ~ 107.45
AUC_0-t_ (ng·h/mL)	64.16	69.71	92.05	18.56	84.71 ~ 100.02
AUC_0-∞_(ng·h/mL)	78.42	82.51	95.04	22.01	86.13 ~ 104.88
**Ezetimibe glucuronide, fed**					
C_max_ (ng/mL)	70.49	72.97	96.60	26.93	85.63 ~ 108.97
AUC_0-t_ (ng·h/mL)	368.83	409.26	90.12	17.03	83.51 ~ 97.25
AUC_0-∞_(ng·h/mL)	489.44	515.02	95.03	20.88	86.56 ~ 104.34
**Total ezetimibe, fed**					
C_max_ (ng/mL)	76.12	78.40	97.10	26.20	86.36 ~ 109.17
AUC_0-t_ (ng·h/mL)	454.24	501.94	90.50	14.12	84.96 ~ 96.40
AUC_0-∞_(ng·h/mL)	520.82	561.0	92.84	18.89	85.32 ~ 101.02

*Abbreviation*: *CI* Confidence interval, *Intra-CV* Intra-subject coefficient of variation *C*_*max*_ Maximum concentration, *AUC*_*0-t*_ The area under the plasma concentration-time curve from 0 h to the time of last measurable concentration, *AUC*_*0-∞*_ The area under the plasma concentration-time curve from 0 h to infinity, *T*_*max*_ Time to reach maximum concentration (Median), *T½* half life

In the fasting study, compared with the reference preparation, the C_max_, AUC_0–t_, and AUC_0-∞_ of ezetimibe of the test preparation was 94.89, 97.56 and 98.09% respectively;the C_max_, AUC_0–t_, and AUC_0-∞_ of ezetimibe glucuronide of the test preparation was 91.3, 96.59 and 95.78% respectively; the C_max_, AUC_0–t_, and AUC_0-∞_ of total ezetimibe (ezetimibe+ ezetimibe glucuronide) of the test preparation was 91.05, 97.14 and 98.13% respectively. These ratios were within the predefined equivalence limit of 80 ~ 125%.These 90% CIs all fell within the range of 80.00% ~ 125.00%.

In the fed study, compared with the reference preparation, the C_max_, AUC_0–t_, and AUC_0-∞_ of ezetimibe of the test preparation was 96.28, 92.05 and 95.04% respectively; the C_max_, AUC_0–t_, and AUC_0-∞_ of ezetimibe glucuronide of the test preparation was 96.60, 90.12 and 95.03% respectively, the C_max_, AUC_0–t_, and AUC_0-∞_ of total ezetimibe (ezetimibe+ ezetimibe glucuronide) of the test preparation was 97.10, 90.50 and 92.84% respectively. These 90% CIs all fell within the range of 80.00% ~ 125.00%.

### Safety

During the whole study period, both ezetimibe tablet and Ezetrol® showed good tolerance. In In the study of fasting condition, a total of 8 volunteers increasing in urinary red blood cells, leukocyte, and neutrophil, sore throat, and epistaxis were observed in 15 times AEs tests. These adverse reactions were considered to be related with the study drugs. In the study of fed condition, 3 volunteers increasing in urine leukocytosis and anemia were detected in 3 times AEs. None of them were judged as serious adverse events (SAEs).

## Discussion

According to FDA guidelines [11], the bioequivalence of ezetimibe tablets in healthy adults was evaluated by 90% CI of ezetimibe and total ezetimibe. In our study, both under fasting and fed conditions, the bioavailability of ezetimibe and total ezetimibe were calculated by C_max_, AUC_0–t_ and AUC_0-∞_, and the 90% CI fell between 80.00–125.00%. The 90% CI calculated by AUC_0-∞_ fell between 0.80–1.25, indicating that the test preparation and the reference preparation are equivalent in absorption degree, metabolism degree and peak concentration. T_max_ of ezetimibe, ezetimibe glucuronide and total ezetimibe were similar in each condition, indicating that absorption rates had no statistically significant difference under fasting condition and fed condition. T_1/2_ and λ_z_, were essentially the same for the two components in both studies.

There is a statistical difference in the incidence of adverse events between the reference and the test preparation, which may be caused by comprehensive factors such as individual differences and environment, but there is no difference in the incidence of adverse reactions. Therefore, it cannot be explained that the two preparations are not equivalent. Ezetimibe tablets have good tolerance and safety under the dose conditions of this study.

These results indicated that the Ezetrol®/ezetimibe tablets were bioequivalent and exchangeable in clinical practice. Therefore, ezetimibe tablet could be further developed to become a convenient option to treat high cholesterol in Chinese patients.

## Conclusions

In conclusion, the test ezetimibe tablets developed by Chongqing shenghuaxi Pharmaceutical Co., Ltd. are equivalent to the Ezetrol® produced by MSD Pharma (Singapore) Pte. Ltd. The results confirmed the PK of ezetimibe tablets to be similar to that of the Ezetrol® after a single dose of 10 mg in healthy chinese participants. The trial proved that ezetimibe tablets and Ezetrol® were bioequivalent under both fasted condition and fed condition. The ezetimibe tablets can be used in the treatment of exogenous cholesterol absorption in the patients.

## Data Availability

All of the data generated or analysed during this study are included in this published article and its supplementary information files.

## Abbreviations

LC-MS/MS: Liquid chromatography-tandem mass spectrometry
GCP: Good clinical practice
CIs: Confidence intervals
NMPA: China National Medical Products Administration
HIV: Human immunodeficiency virus
AEs: Adverse events
AUC: Area under curve
C_max_: Maximum plasma concentration
T_max_: Time to Cmax
T½: Elimination half-life
SD: Standard deviation
BMI: Body mass index
ECG: Electrocardiogram
SAEs: Serious adverse events
